# Management of Airways through Rapid Tracheostomy in a Severely Burnt Patient Attended to via Helicopter

**DOI:** 10.1155/2021/5590275

**Published:** 2021-07-01

**Authors:** Orazio Stefano Giovanni Filippelli, Anna Maria Giglio, Simona Paola Tiburzi, Maria Teresa Archinà, Ercole Barozzi, Pietro Maglio, Stefano Candido, Roberta Viotti, Umberto Riccelli, Mario Pezzi, Carmelo Romano, Anna Maria Scozzafava, Maria Laura Guzzo

**Affiliations:** ^1^Anesthesiology and Intensive Care Department “Pugliese Ciaccio” Hospital, 88100 Catanzaro (CZ), Italy; ^2^Anesthesiology and Intensive Care Department Civil Hospital, 89044 Locri (RC), Italy; ^3^Anesthesiology and Intensive Care Department “San Giovanni di Dio” Hospital, 88900 Crotone (KR), Italy

## Abstract

In Catanzaro, Italy, an adult male with severe burns all over his body and in a state of coma was promptly rescued by the medical team at the air ambulance service (HEMS), who provided airway safety through laryngeal mask placement (LMA). The patient was subsequently transferred to the nearest Hub center, where an emergency tracheostomy was performed to ensure better airway management during the flight to the nearest available major burn center. This is the first documented case at regional level of a patient undergoing rapid tracheostomy through an imminent transfer with air ambulance.

## 1. Rapid Tracheostomy

Among the percutaneous tracheostomy, we include the most widespread: Griggs's Technique and Ciaglia [1] blue Rhino's technique which is most suitable for a patient admitted to the intensive care unit in need of long-term ventilation. Rapid tracheostomy is certainly the best approach to manage the airways of critical patients in these emergency conditions. It can be performed immediately, safely, and with a large margin of success.

This technique involves quickly inserting a small-caliber cannula inside the trachea [2, 3].

For this purpose, it is necessary for the operator to accurately identify the anatomical findings in the neck region corresponding to the structures of the larynx and trachea. With the graphic stationary pen, the upper and the lower margins of the thyroid cartilage are traced on the anterior region of the neck: below the latter, the cricoid cartilage, including the upper margin and the aforementioned lower margin of the thyroid cartilage, is the cricothyroid space; finally, below the cricoid are the first tracheal rings.

The cannula used in this case is mounted on a mandrel-needle at the proximal end of which a syringe is inserted. The technique consists of puncture by means of the spindle of the skin, centrally to the cricothyroid space or under the lower edge of the cricoid cartilage, between the first and second ring once detected at the digital pressure. Cross the layer of the dermis with the mandrel-needle and proceed until the air is sucked into the syringe and connected to it—this then indicates the entry into the trachea. At this point, the long cannula is made to proceed into the mandrel inside the trachea.

## 2. Presentation of the Patient

On November 2019, a 58-year-old man sets himself on fire after dousing himself with petrol inside the city cemetery. After the rescue attempts of those who were present, the patient was taken care of by the basic medical team of the Hospital of Locri (Calabria), who after the positioning of the laryngeal mask (LMA)—due to evident difficulty in proceeding with orotracheal intubation (IOT), transported the patient to the Hub Hospital of Catanzaro, in order to ensure a more complete and safe clinical management, in anticipation of a transfer to the nearest major burn center. The patient who was suffering from a severe form of obesity reported severe third and fourth degree burns [4] in about 95% of the body surface, with evident diffuse edema, especially on the face region, alongside coma (GCS = 3) and diffuse muscle stiffness. At the Hub center, the patient was assisted with 100% O_2_ by means of a Mapleson circuit connected to the LMA, on monitoring the parameters were NIBP 95/50 mmHg, heart rate 120 bmp, and SpO_2_ 92%. Given the high risk of removal of the LMA due to massive edema, particularly in the face, and the high predictive index of difficult intubation, it would have been rather imprudent to remove LMA in order to proceed with orotracheal intubation. Therefore, to secure the airways and arrange for the patient to be transported by air ambulance to the major burn center, it was decided to perform an emergency tracheostomy using the appropriate device. The burns in the neck region rendered the skin near the finding points, of hard consistency, while below the inferior margin of the cricoid, the skin was relatively more elastic.

## 3. Materials and Methods

The emergency tracheostomy device consists the following: cannula 4.0 i.d. with mandrel-needle, demographic pen, Mapleson circuit, mechanical ventilator, triangular-tipped lancet, catheter-mount, optical fibroscope, central venous catheter 7.5 Fr., Lidocaine 2%, Chlorexidine 2%, sterile drapes, and sterile gauze.

The patient was positioned with the head of hyperextension, as far as possible given the cervical rigidity. After the preparation of the operating field by disinfection with Chlorexidine 2% and infiltration of the affected area with 2% Lidocaine, the anatomical landmarks were outlined with a demographic pen.

The mandrel-needle incorporated into the cannula was inserted along the sagittal plane and perpendicular to the skin, 0.5 cm below the lower edge of the cricoid. Once you are sure you are inside the trachea, after the detection of air bubbles through the syringe (containing water) and attached to the mandrel, the cannula needle was made to proceed up to the safety lock. The block was then removed, and the tracheostomy tube was pushed, making it slide along the mandrel towards the cranial-caudal direction, inside the trachea. After the fibroscopic check and the fixing of the cannula flanges with tubular ties, the cannula was connected to the mechanical ventilator by means of a catheter-mount and a mechanical ventilation (VAM) was set in volumetric control mode. The following parameters were set: PEEP 6 cmH_2_O, VTi 600 ml, RFi 15 amp, and FiO_2_ 60%. The LMA was left in situ. During the mechanical ventilation, due to the absence of the cuff on the cannula, the difference between the inspiratory tidal volume (VTi) and the expiratory tidal volume (VTe) was approximately 100 ml. There was a rapid increase in SpO_2_ to 99% and a relative hemodynamic stability.

After placing a central venous access in the right internal jugular vein using ultrasound guidance, 0.9% saline was infused at a rate of 250 ml/h; gauzes soaked in 2% Chlorexidine were applied to the anterior surface of the body. Later the patient was taken to the helipad near the Hub Hospital and transferred to the Severe Burns Center of the Sant'Eugenio Hospital in Rome. After the transfer by helicopter, mechanical ventilation continued with the previously set parameters. During the flight, it was necessary to use the continuous infusion of Norepinephrine 0.05 mcg/kg/min to support hemodynamic stability. The transfer was successful, despite the extremely critical condition of the patient.

## 4. Discussion

In this case, the LMA could not build a definitive solution due to the progressive increase in the edema of the mucous membranes, which would certainly lead to its escape. Moreover, the predictive indices of difficult intubation have led doctors to opt for a quick and safe solution at the same time.

This solution could only be the rapid tracheostomy with a small-caliber cannula that guaranteed immediate access to the trachea and finally optimal management of ventilation in the following moments, especially in view of the immediate transfer to the helicopter.

The presence of tracheostomy proved to be crucial especially in the flight phases, where the patient's clinical stability is essential to minimize any type of intervention in a very limited space such as the cockpit of the AW 109 series, used for the case.

Mechanical ventilation through the cannula without cuff, although of small caliber, proved to be optimal during all phases of transport, with a VT of about 450-550 ml and a peak pressure not exceeding 24 cmH_2_O. This allowed the maintenance of vital parameters within physiological limits, although supported by the continuous infusion of noradrenaline.

## 5. Conclusions

Given the very serious clinical condition of the patient and in order to ensure better management of the airways and achieve stability of the vital parameters, adopting the choice of rapid tracheostomy proved to be decisive for the correct clinical management without complications [5] during the transfer to helicopter, while ensuring the success of the entire operation.

After all, the absence of alternative choices to rapid tracheostomy by the operators who assisted the patient upon his arrival at the hub hospital center is that

1. the clinical and anatomical conditions of the patient with severe burn, in particular to the face with involvement of the mouth and nose, would not have allowed immediate and at the same time safe access to the trachea other than through rapid tracheostomy
2. Furthermore, the transfer via helicopter would not have taken place if the airways had not been secured with a more stable permanent device than the LMA

Other solutions, such as the surgical approach, would not have had the characteristics of immediacy and would not have been free from more serious complications due to the conditions in which the tissues were present.

Finally, the helicopter flight to the center for severe burns has the characteristics of a secondary transfer, that is, an assisted transfer of a patient whose clinical conditions make it significantly preferable to transport by helicopter over road transport, since the prognosis depends on the time taken to its success.

The feasibility of the mission is always subject to the presence of the therapeutic conditions put in place for the purpose of its success; therefore, rapid tracheostomy was the best possible approach in this case.

## Data Availability

Archive of medical records of the emergency room is from Hospital “Pugliese-Ciaccio,” Catanzaro; Regional rescue mission archive, Catanzaro Operations Centre; Register of Tracheostomies, Anesthesia-Resuscitation and Intensive Care Unit, “Pugliese-Ciaccio” Hospital, Catanzaro.
